# Micro-Mechanical Properties and Deformation Damage Behavior of the Matrix and Primary Carbides in 8Cr4Mo4V Bearing Steel

**DOI:** 10.3390/mi17010113

**Published:** 2026-01-15

**Authors:** Chenhui Sun, Xubo Fan, Xiaoquan Shi, Junjun Liu, Zhihu Zhang, Bohan Zhang, Haitao Liu

**Affiliations:** Department of Mechanical Engineering and Automation, Harbin Institute of Technology, Harbin 150001, China; hdsunchenhui@163.com (C.S.); 13846216963@163.com (X.F.); 24s108275@stu.hit.edu.cn (J.L.); 13375837726@163.com (Z.Z.); klee@hit.edu.cn (B.Z.)

**Keywords:** 8Cr4Mo4V bearing steel, primary carbides, mechanical properties, deformation damage, nanoindentation, nano-scratch

## Abstract

8Cr4Mo4V bearing steel is a critical material for main shaft bearings in aero-engine applications. However, the current understanding of the micro-mechanical properties of its matrix and primary carbide phases (vanadium-rich and molybdenum-rich carbides) remains insufficient. This knowledge gap readily induces various forms of deformation damage during grinding, severely compromising the surface integrity of the workpiece. To address this, nanoindentation and nano-scratch techniques were employed to systematically quantify the micro-mechanical properties of each phase and investigate the deformation damage behavior of the steel under load. Results showed that MC carbides exhibited the highest elastic modulus and microhardness, which made them more susceptible to becoming crack initiation sites during grinding. Nano-scratch testing further revealed that crack initiation at carbide edges and localized spalling were the primary damage mechanisms. This study provides a micro-mechanical foundation for controlling the grinding surface quality of 8Cr4Mo4V bearing steel, holding significant implications for optimizing grinding processes, suppressing crack initiation, and elucidating the grinding damage mechanism.

## 1. Introduction

8Cr4Mo4V bearing steel is recognized as the second-generation high temperature bearing steel, derived from high-speed steel. This material exhibits outstanding high-temperature resistance and fatigue endurance, making it an ideal material for the main shaft bearings in aero-engine applications [1,2,3]. For the manufacturing of these high-precision components, grinding is employed as the final forming process, and its quality directly determines the surface integrity of the workpiece [4,5]. However, most of the research concerning 8Cr4Mo4V bearing steel has predominantly focused on its service-related behavior, such as fatigue [6], wear [7], and corrosion [8]. In contrast, limited fundamental studies have addressed its critical grinding process. This gap stems from an insufficient understanding of the mechanical properties and associated deformation damage behavior of its constituent microstructures. This understanding is, critically, a prerequisite for elucidating the grinding damage mechanism.

8Cr4Mo4V bearing steel contains multiple alloying elements (Mo, V, Cr, etc.), which precipitate to form diffusely distributed primary carbide phases during high-temperature diffusion processes [9,10]. To date, several studies have explored the mechanical characteristics of these phases. For instance, Lu et al. [11] predicted the hardness of carbides in iron-based multi-element alloys using different models based on bonding characteristics. Casellas et al. [12] evaluated the fracture toughness ($K_{c}$) of primary carbides in tool steels. Furthermore, Lin et al. [13] assessed mechanical properties such as hardness and toughness of primary carbides in high-carbon chromium-based steels. Using in situ tensile testing, Liu et al. [14] discovered that M_2_C (molybdenum-rich carbides) carbides exhibit lower fracture strength and are therefore more susceptible to fracture. Yang et al. [15] investigated the influence of primary carbide size in M50 bearing steel on sliding wear and rolling contact fatigue behavior, demonstrating superior wear resistance for MC (vanadium-rich carbides) carbides. Although the presence of these primary carbides is known to enhance the overall mechanical properties of the steel [7,9], their intrinsic micro-mechanical properties and the performance disparities between them and the matrix remain understudied. Moreover, how this performance difference governs damage behavior during grinding remains unclear, hindering the optimization of grinding parameters for specific microstructural regions.

Among various machining methods, grinding represents a process that balances both high precision and efficiency [16,17,18,19]. Nevertheless, because the grinding process is inherently accompanied by high strain rates and significant plastic deformation, defects such as microcracks, particle detachment, and plastic flow readily occur on the workpiece surface or subsurface [4,18,19,20,21]. Previous studies have characterized this complexity in diverse materials. For instance, Zhang et al. [22] conducted form grinding experiments on 20Cr2Ni4A alloy steel gears, demonstrating that variations in grinding parameters lead to non-uniform surface roughness distribution. Chen et al. [23] analyzed the initiation and propagation behavior of grinding cracks in 40Cr steel camshafts from multiple perspectives. Furthermore, Tan et al. [24] investigated the surface integrity of GCr15 bearing steel under different grinding methods, concluding that free abrasive grinding yields a more intact surface under identical conditions. Zhao et al. [25] investigated the effect of shape adaptive grinding on the surface integrity of ductile cast iron. Kaplonek et al. [26] investigated the grinding process of nickel-chromium-based superalloy and reduced chip clogging and friction during grinding by improving the grinding wheel composition. These studies collectively highlight the complexity of grinding damage across diverse materials and processes. Therefore, to effectively suppress damage in 8Cr4Mo4V bearing steel during grinding, it is imperative to explicitly elucidate its deformation and damage mechanisms under high-strain-rate grinding action. This investigation will provide a crucial theoretical foundation for enhancing the surface integrity of this critical component.

To this end, this study employs nanoindentation and nano-scratch techniques to quantify the micro-mechanical properties of the 8Cr4Mo4V steel matrix and its primary MC and M_2_C carbides. This is achieved by analyzing the micro-morphology of indentations and load–displacement curves, thereby elucidating their deformation and damage behavior under simulated high-strain-rate grinding conditions. The ultimate goal of this work is to provide the essential micro-mechanical foundation for controlling the grinding surface quality of 8Cr4Mo4V bearing steel. It, therefore, holds significant implications for subsequent grinding process optimization, crack initiation suppression, and the elucidation of the grinding damage mechanism.

## 2. Materials and Methods

### 2.1. Materials

The material used in this study was 8Cr4Mo4V bearing steel. It was subjected to a specific heat treatment process: nitriding quenching at 1150 °C, followed by deep cryogenic treatment at −30 °C, and finally three cycles of nitriding tempering at 550 °C. The primary chemical composition of the steel is listed in Table 1.

The dimensions of the 8Cr4Mo4V bearing steel workpiece are 20 mm × 14 mm × 5 mm. For microstructural observation and subsequent mechanical testing, the samples were sequentially ground using 800#, 1000#, 1500#, and 2000# emery papers. This was followed by fine polishing using a metallographic polishing machine (Mingzheng (Zhejiang) Electronic Equipment Co., Ltd., Jiaxing, China). Finally, the surface was etched for 30 s using a 5% nitric acid in alcohol solution.

### 2.2. Characterization of Micro-Mechanical Properties

**Figure 1:**
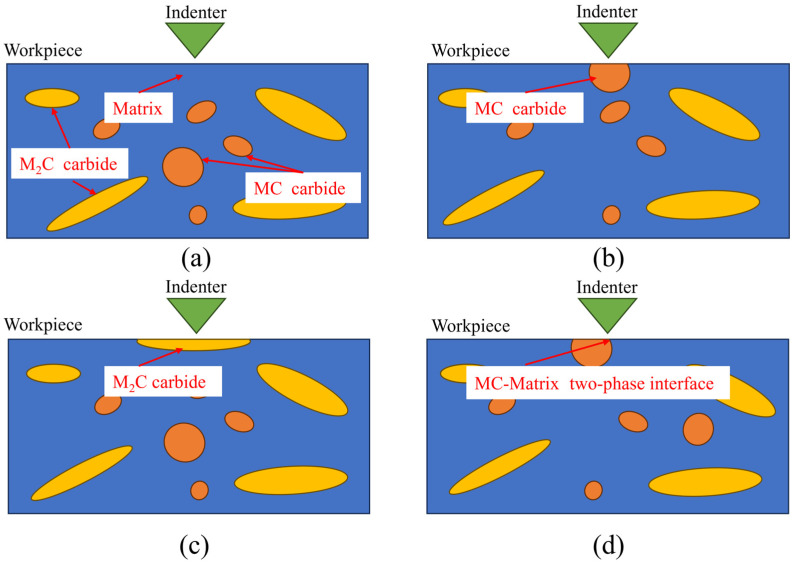
Schematic Illustration of the Nanoindentation Experiment: (**a**) Matrix; (**b**) MC carbide; (**c**) M_2_C carbide; (**d**) MC-Matrix two-phase interface.

Micro-mechanical property characterization of the 8Cr4Mo4V bearing steel matrix and its primary MC and M_2_C carbides was performed using nanoindentation, executed on a KLA Instruments™ Nano Indenter^®^ G200X (Milpitas, CA, USA) equipped with a Berkovich indenter. Nanoindentation experiments were specifically conducted on the 8Cr4Mo4V steel matrix phase, MC carbide, M_2_C carbide, and the interface region between the MC carbide and the matrix. The testing configuration is schematically illustrated in Figure 1a–d. The indentation depth was set to 300 nm to ensure the indentation area did not exceed the size of the carbides and a dwell time of 25 s at the maximum load to account for potential creep effects. All experiments were conducted at room temperature. During the nanoindentation process, the apparatus automatically recorded the load and displacement variations, generating a load–displacement (P–h) curve. This curve was then used to directly obtain the maximum indentation depth (h_max_) and the maximum load (P_max_).

The load–displacement (P–h) curves for 8Cr4Mo4V bearing steel matrix, MC carbide, M_2_C carbide, and the MC-matrix two-phase interface were obtained by continuously recording the indenter displacement versus load relationship throughout a complete loading and unloading cycle. Based on the acquired load–displacement (P–h) curves, the elastic modulus (E) and microhardness (H) of the respective microstructural constituents were calculated.

Hardness (H) is defined as the maximum load (P_max_) divided by the projected contact area (A_c_) of the indentation [27,28]. It is calculated using the following Equation (1):

(1) $$H=\frac{P_{max}}{A_{c}},$$

where P_max_ is the maximum applied load, and A_c_ is the projected contact area of the indenter.

The elastic modulus (E) of the material is determined by the following Equation (2):

(2) $$E=(1-\nu^{2})(\frac{1}{E^{*}}-\frac{1-\nu_{i}^{2}}{E_{i}}{)}^{-1},$$

where *E* and ν are the elastic modulus and Poisson’s ratio of the material, respectively; *E_i_* and ν*_i_* are the elastic modulus and Poisson’s ratio of the indenter, respectively; and $E^{*}$ is the reduced modulus.

The fracture toughness (K_c_) of a material cannot be determined directly from the load–displacement (P–h) curve, but it can be calculated using a specific Equation (3) [29].

(3) $$K_{c}=\lambda (\frac{E}{H}{)}^{1/2}\frac{P}{c^{3/2}},$$

where λ is an empirical constant, which is taken as 0.016 for the Berkovich indenter; and *c* is the total length of the radial crack and the half-diagonal of the indentation.

### 2.3. Investigation of Material Deformation and Damage Behavior

**Figure 2:**
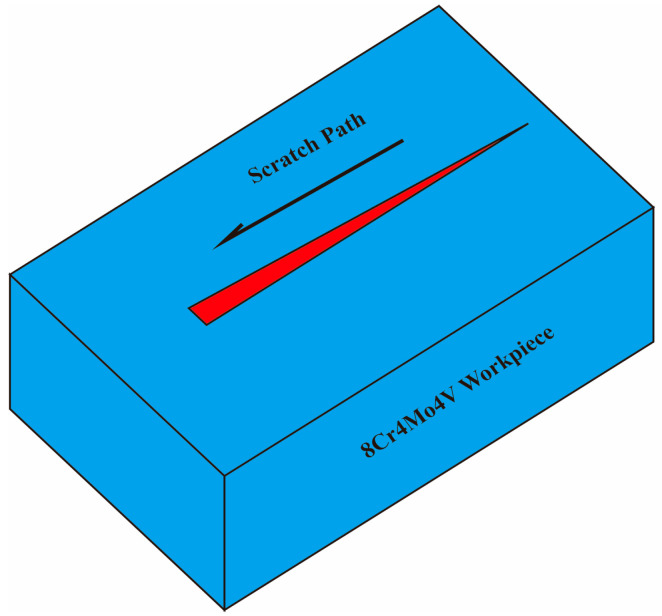
Schematic Illustration of the Nano-scratch Process.

To investigate the material deformation and damage behavior of 8Cr4Mo4V bearing steel during grinding, the nano-scratch method was employed to simulate the single-abrasive-grain grinding process. This test was performed using a KLA Instruments™ Nano Indenter^®^ G200X equipped with a Berkovich indenter. The Berkovich indenter was oriented facing the scratch direction, with applied loads of 25 mN and 50 mN, a scratch length of 40 μm, and a loading rate of 200 mN/s. The scratch path was planned to traverse both the matrix and primary carbides. All experiments were conducted at ambient temperature. A schematic illustration of the nano-scratch process is shown in Figure 2.

## 3. Results and Discussion

### 3.1. Calculation Result

Based on nanoindentation experiments and the derived calculation formulas, the elastic moduli (E) and microhardness (H) values for the 8Cr4Mo4V bearing steel matrix, MC carbide, and M_2_C carbide were calculated using Equations (1) and (2). The specific results are summarized in the corresponding Table 2. The results showed that the tempered 8Cr4Mo4V matrix exhibited an elastic modulus of approximately 238.431 GPa and microhardness of 7.736 GPa. The MC carbides, in contrast, demonstrated the highest values, with an elastic modulus of approximately 353.420 GPa and microhardness of 21.703 GPa. The M_2_C carbides had an elastic modulus of approximately 289.683 GPa and microhardness of 13.463 GPa. By comparing the current limited number of measurement data, it can be observed that the elastic modulus and microhardness of the primary carbides are generally higher than those of the matrix. This significant trend of difference can be attributed to the strengthening effect of alloy elements Mo and V. Therefore, the data from this study should mainly be regarded as preliminary evidence revealing the performance difference trends between the various phases. This substantial disparity in mechanical properties between the primary carbides and the matrix implies that the selection and control of process parameters during machining critically affect the quality and integrity of the machined surface.

During the nanoindentation testing, cracks of varying lengths were observed in both the MC and M_2_C carbides. Therefore, to evaluate the resistance to crack propagation of these primary carbides during the grinding process, the fracture toughness (K_c_) of the respective carbides was calculated using Equation (3).

After calculation, the fracture toughness (K_c_) of 3.7 MPa m^1/2^ for the MC carbides and 2.2 MPa m^1/2^ for the M_2_C carbides. This difference demonstrates that the presence of finer and uniformly distributed MC carbide particles enhances the overall toughness of the 8Cr4Mo4V bearing steel, and, simultaneously, the MC carbides exhibit superior resistance to crack propagation and material fracture.

### 3.2. Micro-Mechanical Properties of Individual Microstructures in 8Cr4Mo4V Bearing Steel

**Figure 3:**
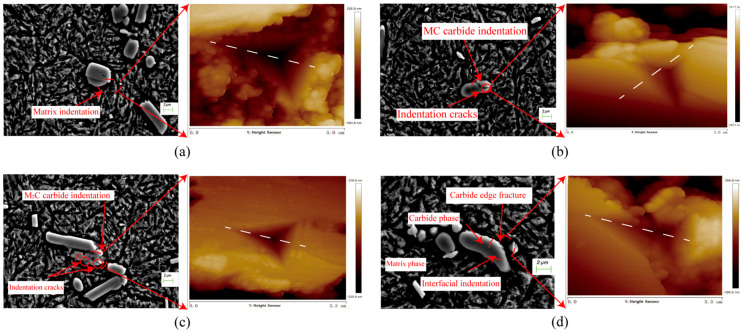
SEM and AFM Morphologies of Individual Microstructures in 8Cr4Mo4V Bearing Steel: (**a**) Matrix; (**b**) MC carbide; (**c**) M_2_C carbide; (**d**) MC-Matrix two-phase interface.

The post-indentation micro-morphologies of the matrix, MC carbide, M_2_C carbide, and MC-matrix two-phase interface were characterized using scanning electron microscopy (SEM) and atomic force microscopy (AFM), as illustrated in Figure 3a–d. Figure 3a shows that when the load was applied to the matrix material, no significant fracture was observed, pronounced pile-up was evident. In contrast, Figure 3b,c demonstrate that when the load was applied to the MC and M_2_C carbide particles, respectively, both exhibited distinct fracture phenomena accompanied by crack formation, with material piling being less pronounced. Figure 3d presents the SEM and AFM three-dimensional topography of the MC-matrix two-phase interface region. It is evident that the carbide edges experienced a degree of fracture, which was accompanied by crack initiation and propagation, alongside some material accumulation.In the subsequent measurements, we will use the dashed lines in the figure as a reference, collect data from various locations, and thereby plot the cross-sectional profile of the nanoindentation.

#### 3.2.1. Mechanical Properties of the Matrix

**Figure 4:**
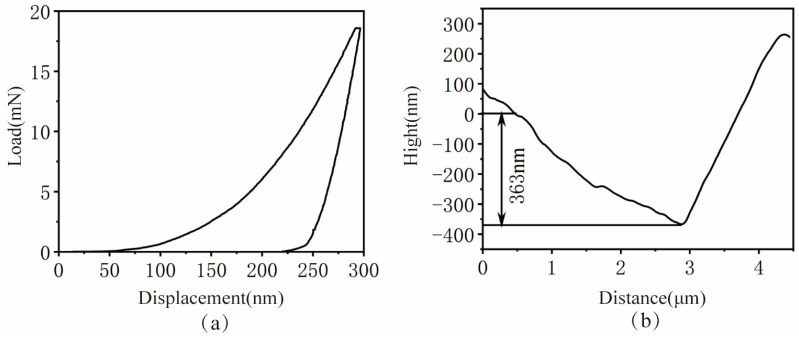
Nanoindentation Results of the Matrix: (**a**) Load–displacement (P–h) curve; (**b**) Nanoindentation cross-sectional profile.

Figure 4a displays the load–displacement (P–h) curve obtained during the matrix nanoindentation process. The graph reveals that the applied load exhibited an approximately linear increase during the loading phase. Once the predetermined maximum indentation depth was reached, the unloading procedure was initiated, and the material’s elastic recovery commenced. Significantly, a hysteresis loop exists between the loading and unloading curves of the matrix, which clearly demonstrates that plastic deformation occurred in the material during the nanoindentation process.

Figure 4b displays the contour curve of the matrix nanoindentation cross-section. The figure reveals that the pile-up phenomenon in the matrix material was more pronounced, which indicates that the matrix possesses high plasticity. Notably, although the maximum indentation depth was set at 300 nm in this experiment, the actually measured depth was 363 nm. Measuring discrepancy stems from the inherent uncertainties in the instrument’s leveling process and zero reference surface determination when conducting scans on micrometer-scale indentations, as well as the interference from the surface topography around the indentations. These factors jointly affect the absolute accuracy of the depth measurement. This characteristic high plasticity of the matrix material is expected to result in significant pile-up, material protrusions, and traces of plastic flow on the machined surface during grinding operations.

#### 3.2.2. Mechanical Properties of the MC Carbide

**Figure 5:**
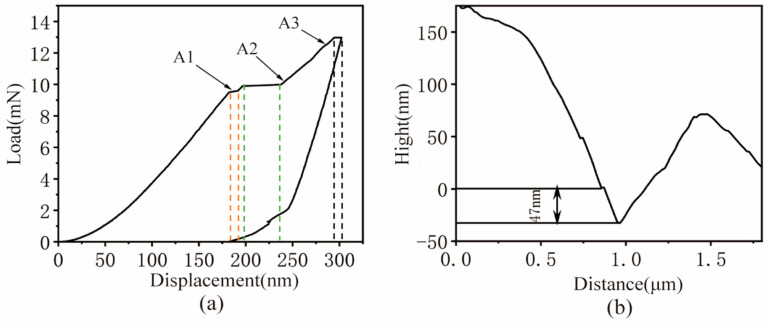
Nanoindentation Results of the MC carbide: (**a**) Load–displacement (P–h) curve; (**b**) Nanoindentation cross-sectional profile.

Figure 5a displays the load–displacement (P–h) curve obtained during the nanoindentation of the MC carbide. The curve exhibits three clear inflection points, denoted as A1, A2, and A3. The dashed lines of different colors in the figure correspond to the load plateau stages repeatedly generated under different indenter displacements. This suggests that prior to A1 (at an indentation depth of approximately 170 nm), the load increased rapidly with increasing indentation depth. During the stage from A1 to A2, the load remained relatively constant, forming a distinct load plateau. This indicates that microscopic cracks have already formed and broken within the MC carbides. This phenomenon can be attributed to the fragmentation of the carbides along the grain boundaries. Therefore, during the continuous pressing process by the indenter, the load did not increase with the increase in depth, resulting in the observed load plateau. Beyond point A2, the load resumed its rapid increase with further penetration until point A3, where the P–h curve repeated the A1–A2 pattern. This behavior demonstrates the continuous fracturing of the MC carbide as the load increases. This pronounced phenomenon implies that during the actual grinding process, substantial grinding forces could induce repeated microfracture of the MC carbides, leading to the nucleation and propagation of numerous microcracks and, consequently, severe grinding damage.

Figure 5b displays the contour curve of the nanoindentation cross-section for the MC carbide. Although there are limitations in the absolute measurement accuracy of the cross-sectional profile at the nanoscale, the high-resolution morphological information it provides has become the most intuitive evidence for directly observing and revealing the material accumulation phenomenon around indentations. As is evident from the figure, following elastic recovery, the residual indentation depth in the MC carbide was only 47 nm, indicating its high elastic recovery capability. This characteristic consequently renders it more susceptible to brittle damage during grinding operations, such as microcracks and particle spalling.

#### 3.2.3. Mechanical Properties of the M_2_C Carbide

**Figure 6:**
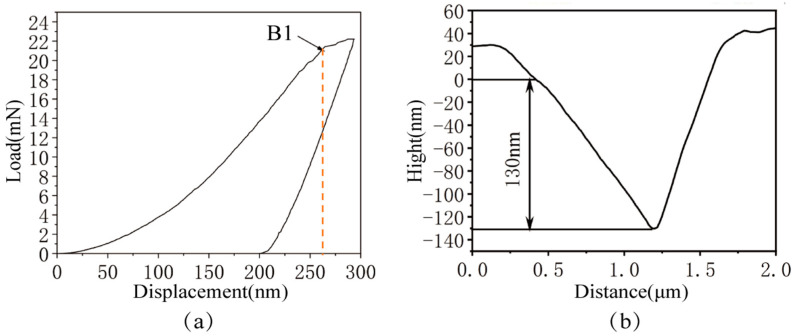
Nanoindentation Results of the M_2_C carbide: (**a**) Load–displacement (P–h) curve; (**b**) Nanoindentation cross-sectional profile.

Figure 6a displays the load–displacement (P–h) curve obtained during the nanoindentation of the M_2_C carbide. The curve indicated that as the indenter penetrated the M_2_C carbide, the load exhibited a rapid increase with increasing indentation depth. However, when the indentation depth reached approximately 250 nm, near point B1, the rate of load increased markedly slowly. It is therefore inferred that at an indentation depth of approximately 250 nm, cracking or localized fracture occurred within the M_2_C carbide, leading to a change in the contact behavior between the indenter and the material.

Figure 6b displays the contour curve of the M_2_C carbide nanoindentation cross-section. Although there are certain precision limitations, it can be clearly seen from the cross-sectional contour curve that after the elastic recovery process, the residual indentation depth of the M_2_C carbide measured 130 nm, representing a moderate residual depth. This indicates that M_2_C carbide possesses moderate elastic recovery capability and moderate plasticity. Consequently, it is inferred that, compared to MC carbide, M_2_C carbide will exhibit a greater degree of plastic behavior during grinding operations. This is expected to result in plastic deformation and localized material accumulation, leading to the formation of a certain quantity of micro-pits and plastic flow traces on the machined surface.

#### 3.2.4. Mechanical Properties of the MC-Matrix Two-Phase Interface

**Figure 7:**
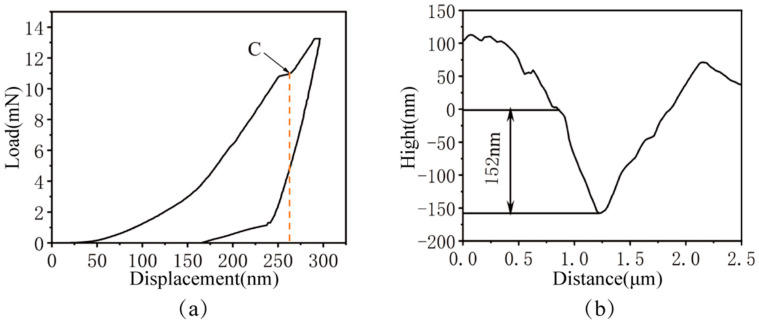
Nanoindentation Results of the MC-Matrix Two-Phase Interface: (**a**) Load–displacement (P–h) curve; (**b**) Nanoindentation cross-sectional profile.

The cross-sectional transition zone between the primary carbide and the matrix phase is narrow, typically measuring 1 nm to 20 nm in width. This inherently results in the mechanical properties at the phase interface being influenced by both the matrix and the carbide simultaneously. Figure 7a displays the load–displacement (P–h) curve obtained during nanoindentation of the MC-matrix two-phase interface. As can be seen from the figure, the mechanical properties of the MC-matrix interface are jointly influenced by both the matrix and the MC carbide. The curve revealed that as the interface approached the two-phase interface, the load increased with penetration depth. Upon reaching approximately 250 nm penetration depth, a distinct inflection point (point C) emerged, after which the rate of load increase slowed. Subsequently, the unloading procedure was initiated, and the material’s elastic recovery began.

Based on the calculated mechanical properties of the matrix and MC carbides, it is inferred that during nanoindentation at the MC-matrix two-phase interface, the initial response involves the brittle fracture of the MC carbide. However, the synergistic deformation and constraint effect of the surrounding matrix material suppress the early initiation and propagation of cracks. Consequently, the penetration depth at which brittle fracture occurs in the MC carbide is significantly increased compared to the pure MC carbide. Subsequently, the reduction in load-bearing capacity caused by this brittle fracture leads to the two-phase interface to progressively exhibit the mechanical characteristics of the surrounding matrix material.

Figure 7b displays the nanoindentation cross-sectional profile curve at the MC-matrix two-phase interface. The figure revealed that following elastic recovery, the residual indentation depth measured 152 nm, accompanied by substantial material accumulation. This result further indicates that the presence of the MC phase enhances the overall toughness of the 8Cr4Mo4V bearing steel; however, the efficacy of MC in mitigating matrix material accumulation warrants further investigation. Future work can be identified through the implementation of measures such as large-scale scanning that includes indentations and the entire surrounding surface.

### 3.3. Deformation and Damage Behavior of 8Cr4Mo4V Bearing Steel

**Figure 8:**
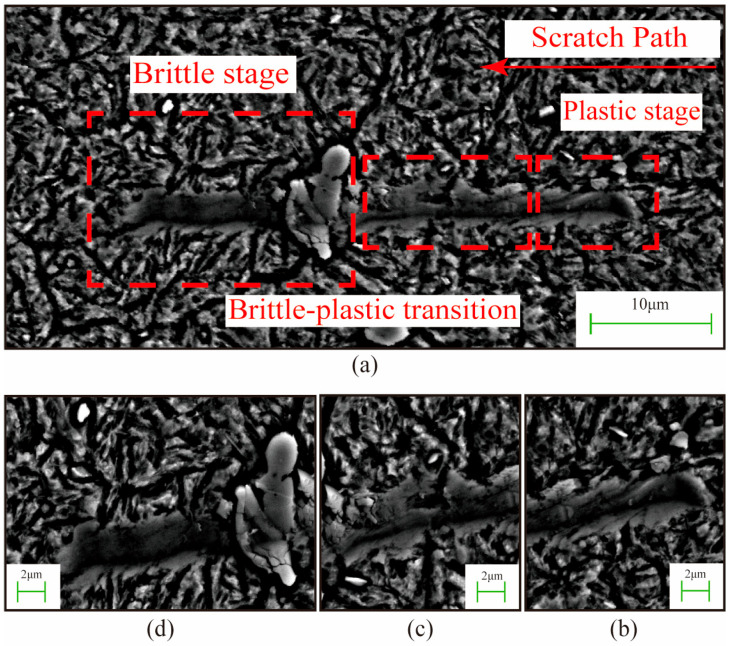
SEM Morphology of Nano-scratch on 8Cr4Mo4V Bearing Steel under 25 mN Load: (**a**) Overall view; (**b**) Plastic removal stage; (**c**) Brittle-ductile transition stage; (**d**) Brittle removal stage.

The nano-scratch test is essentially a micro-grinding process, which can be viewed as a single-diamond material removal procedure. Through this test, it is possible to more realistically simulate the material deformation and removal mechanism caused by abrasive particles during the grinding process [30]. Figure 8 presents the SEM image of a nano-scratch on 8Cr4Mo4V bearing steel under a 25 mN load. The image revealed that the material underwent three primary removal modes during nano-scratching: a plastic removal stage, a brittle-ductile transition stage, and a brittle removal stage. Upon the indenter’s initial contact with the matrix material, no significant cracks or particle spalling were observed on either side of the scratch. However, material bulging and accumulation were evident on both sides, indicating that plastic removal occurred during the matrix indentation. This process involved localized material extrusion and flow, resulting in plastic damage. As the indenter gradually approached the primary carbide from the matrix material, distinct cracks developed near the carbide edges. If the bond between the primary carbide and the matrix is weak, carbide detachment occurs, which consequently degrades surface topography quality and compromises surface integrity. During this stage, the material removal mechanism progressively shifted from plastic deformation to brittle fracture. The image clearly revealed fracturing and localized spalling of the primary carbide, leading to brittle damage.

Furthermore, a comparison of the nano-scratch results obtained under 25 mN and 50 mN loads reveals that the load magnitude significantly influences the material removal mode and surface damage. Under a lower load of 25 mN, the material exhibits a typical plasticity-dominated removal mechanism, and the morphology of the scratch shows a continuous and uniform groove structure, with an average width of 2.4 ± 0.3 μm and a depth of 0.2 ± 0.1 μm. However, when the load is increased to 50 mN, the material’s removal mechanism changes, showing characteristics of a brittle-plastic mixed damage, with the scratch width increasing to 6 ± 0.5 μm and the depth reaching 1.2 ± 0.3 μm. This indicates that at lower loads, the material exhibits a typical plastic-dominated damage behavior; however, with increasing load magnitude, the surface damage progressively transitions from plastic damage to a brittle-ductile mixed-mode damage mechanism.

By comparing the nano-scratch responses under 25 mN and 50 mN loads, it is suggested that the actual grinding process should be optimized in stages. That is, during the coarse grinding stage, a higher single abrasive particle load may lead to carbide fragmentation and subsurface damage. At this time, the focus of process design should be on controlling the depth of the damage layer. However, in the fine grinding stage, it is recommended to reduce the processing load, thereby promoting a material removal mode mainly based on plastic deformation, reducing carbide fragmentation, and maintaining the integrity of the processed surface.

## 4. Conclusions

In this study, nanoindentation and nano-scratch testing were employed to evaluate the mechanical properties of the individual microstructural constituents of 8Cr4Mo4V bearing steel and to investigate its deformation and damage behavior during grinding. Consequently, a micro-mechanical foundation for the grinding process of 8Cr4Mo4V bearing steel has been established. This foundation holds critical significance for optimizing grinding processes, suppressing crack initiation, and elucidating damage mechanisms. The principal conclusions drawn from this research are summarized as follows:

(1) Significant differences in the micro-mechanical properties of the 8Cr4Mo4V bearing steel matrix, MC carbide, and M_2_C carbide have been quantified. The MC carbide exhibits significantly higher elastic modulus (E) and microhardness (H) values than both the matrix and M_2_C carbide. Conversely, the MC carbide demonstrates greater susceptibility to crack initiation, functioning as a primary crack source. This substantial disparity in mechanical properties among the phases constitutes a primary cause of surface quality deterioration during grinding operations.
(2) The mechanical properties at the MC-Matrix two-phase interface are influenced by the dual effects of both the matrix and the carbide. The presence of the MC phase significantly enhances the toughness of 8Cr4Mo4V bearing steel, and the synergistic deformation between the matrix and MC carbide effectively inhibits the early initiation and propagation of cracks at the MC edges.
(3) During the grinding process, 8Cr4Mo4V bearing steel undergoes three distinct material removal stages: plastic removal, brittle-ductile transition, and brittle removal. Noticeable cracks form along the carbide edges, accompanied by localized spalling of carbide particles, which collectively constitutes the primary form of deformation damage incurred during grinding.

Finally, the micro-mechanical property characterization and processing damage analysis method established in this study can be extended and applied to the study of damage mechanisms in other multi-phase alloys containing hard brittle phases during high-precision processing. At the same time, the precise mechanical parameters of each phase obtained through experimental quantification in this study also provide key input data for the subsequent construction of cross-scale computational models. Based on such models, in the future, it will be possible to theoretically predict and optimize the formation process and damage distribution of complex surfaces under different grinding process parameters.

## Figures and Tables

**Table 1:** Chemical Composition of the 8Cr4Mo4V Bearing Steel (wt.%).

C	Cr	Mo	V	Si	Mn	S	Fe
0.75~0.85	3.75~4.25	4.00~4.50	0.90~1.10	≤0.25	≤0.35	≤0.20	balance

**Table 2:** Results of Elastic Modulus and Microhardness for Each Microstructural Constituent.

Microstructural Constituent	Number	Elastic Modulus (GPa)	Microhardness (GPa)
Matrix	1	235.744	7.863
	2	241.118	7.609
MC carbide	3	345.036	21.205
	4	361.803	22.201
M_2_C carbide	5	288.048	13.259
	6	291.318	13.667

## Data Availability

The data presented in this study is available on request from the corresponding authors.
